# Comparative observation of common tracers in sentinel lymph node biopsy of breast cancer and a study on simplifying its surgical procedure

**DOI:** 10.3389/fsurg.2023.1180919

**Published:** 2023-05-15

**Authors:** Shuo Sun, Jiming Bai, Xiang Wang

**Affiliations:** Department of Thyroid and Breast Surgery, Chengde Central Hospital, The Second Clinical Hospital of Chengde Medical College, Chengde, China

**Keywords:** breast cancer, sentinel lymph node biopsy, tracer, operation procedure, lymphatic vessel

## Abstract

**Background:**

Many breast cancer patients have avoided axillary lymph node dissection after sentinel lymph node biopsy (SLNB). During the SLNB operation, the color of lymphatic vessels is sometimes poor and so finding them is difficult. This study observed the tracing effects of three tracer combinations and also reported our experience in simplifying the SLNB program.

**Methods:**

In total, 123 breast cancer patients whose TNM stage was cT1–2N0M0 were retrospectively studied. According to the tracer used, the patients were divided into the carbon nanoparticle (CNP) group (38 cases), CNP combined with methylene blue (CNP + MB) group (41 cases), and indocyanine green combined with MB (ICG + MB) group (44 cases). All 123 breast cancer cases were also classified into the non-tracking group (53 cases) and tracking group (70 cases) according to the SLNB operation process. The non-tracking group looked for the stained sentinel lymph nodes directly, while the tracking group looked for the stained lymph nodes along the lymphatic vessels.

**Results:**

The SLN identification rates in the CNP, CNP + MB, and ICG + MB groups were 97.4%, 97.6%, and 95.5% respectively (*P* > 0.05). The average number of SLNs detected was 4.92 ± 2.06, 5.12 ± 2.18, and 4.57 ± 1.90, respectively (*P* > 0.05). The ideal display rates of lymphatic vessels in the three groups were 86.8%, 87.8%, and 93.2%, respectively (*P* > 0.05). The SLN identification rates in the non-tracking and tracking groups were 96.2% and 97.1%, respectively (*P* > 0.05). The average number of SLNs detected were 5.73 ± 1.76 and 5.70 ± 1.93, respectively (*P* > 0.05), and the average operation time was 16.47 ± 5.78 and 27.53 ± 7.75 min, respectively (*P* < 0.05).

**Conclusion:**

This is the first study to observe the application effect of CNP combined with MB and ICG combined with MB tracers in SLNB of breast cancer patients. No significant difference was observed among the patients in SLN identification and lymphatic vessel display. Omitting the step of searching for lymphatic vessels in SLNB surgery does not reduce the surgical effect, but the reduced operating steps can reduce the surgical time and theoretically reduce postoperative complications.

## Introduction

The recurrence and survival rates of breast cancer largely depend on the involvement of the axillary lymph node (ALN) and its extent of involvement. Axillary lymph node dissection (ALND) was previously identified as the standard axillary treatment for breast cancer (1, 2). ALND should be performed even for early breast cancer, because it can reduce the recurrence risk and improve the survival rate (3, 4). However, ALND-induced complications are very common, such as axillary lymphedema, limited activity, stiffness, numbness, and serosa formation, and the risk of vascular and brachial plexus injury also exists (5, 6). Lately, ALND for early breast cancer patients with clinically negative lymph nodes (cN0) did not further improve the survival rate or prolong the survival period, but patients experienced unnecessary postoperative complications (5, 7–10). Therefore, sentinel lymph node biopsy (SLNB) has been recently investigated, and in some cases, SLNB is used as an alternative to ALND.

SLNB was used to assess the ALN status of cN0 patients (11, 12). Since its introduction, many changes have been implemented in the treatment of armpits of breast cancer patients. Currently, ALND can be avoided not only in SLN-negative cN0 patients (13, 14) but also in SLN-positive patients receiving breast radiotherapy, axillary radiotherapy, or a combination of both, as long as the number of SLN-positive cases is not more than 2, and no obvious axillary nodules are observed during palpation (15–17). In addition, patients with clinical lymph node-positive (cN+) who were ycN0 after neoadjuvant chemotherapy (NAC) can use SLNB instead of ALND (18, 19).

Breast SLN is the first lymph node to receive lymphatic drainage from the breast and has the highest probability of being affected by cancer cells. If SLN exhibits no metastasis, the probability of ALN metastasis is very low. Clearly displayed lymph nodes under direct vision are the most ideal for surgeons during surgery. With the use of ideal tracers and identification methods, lymphatic vessels and SLN can be clearly displayed during surgery. In 1977, Cabanas (20) first successfully drew the SLN map of patients with penile cancer by using lymphographic and radioimaging agents. Since then, a continuous improvement has been made in the tracer and the equipment required for its identification. At present, dyes commonly used for SLN tracing of breast cancer in the clinic include blue dye methylene blue (MB), patent blue, black dye carbon nanoparticle (CNP), fluorescent dye indocyanine green (ICG), radioisotope 99mTc sulfur colloid, etc. Although the identification rate of fluorescent dyes such as ICG and radioisotopes is high, they can only be observed using instruments after surgical incision, which is not as convenient when visualized with under the ordinary light. Therefore, these dyes are often used in combination with the cheap MB. Many studies have reported the effects of tracers, including those applied singly and in combination or a comparison of the effects of tracers (21–23). However, reports on the effect of comparative observation of CNP and ICG + MB are lacking. This study retrospectively analyzed the tracking effect and safety of SLNB in 123 breast cancer patients who were divided into three groups: CNP, CNP plus MB, and ICG plus MB.

According to the clinical practice guidelines for SLN biopsy for early breast cancer patients issued by the Chinese Society of Breast Surgery (24), SLN should be found along the lymphatic vessels when performing lymph node biopsy. However, in the actual operation, we found that the stained lymphatic vessels were much concealed or not stained in some cases, and finding the stained lymphatic vessels was difficult.

Regarding the display of lymphatic vessels, the ICG-emitted infrared light can penetrate a limited thickness of the human tissue, and the route of lymphatic drainage and the lymph nodes can be observed using the infrared detector. Ye (25) explored the ICG concentration with a series of dilution and found that the display rate of subcutaneous lymphatic vessels at the optimal concentration of 0.723 mg/ml was 80%. The CNP dye alone can dye the lymphatic vessels, but as it passes through the lymphatic vessels and enters the lymph nodes quickly, it stays in the lymphatic vessels for a short period. Surgeons need a large amount of training to complete SLNB. The combination of CNP or ICG and MB can exhibit their respective advantages and improve accuracy.

According to our experience, regardless of whether the stained lymph vessels are seen or not, the stained lymph nodes can be observed after they are separated from the lateral edge of the pectoralis major muscle, which can achieve a good SLNB effect. This will save the time of surgery, reduce the difficulty of operation, reduce the occurrence of surgical complications, and will not allow missing of lymph nodes. This paper also reported the results of this simplified operation on SLNB.

## Materials and methods

### Research object

From November 2017 to December 2022, 123 primary breast cancer patients in our hospital were diagnosed as having breast cancer on the basis of the pathological results of preoperative puncture or intraoperative frozen section pathology. The clinical TNM stage was determined according to the results of physical examination, breast and ALN ultrasound, mammography, breast MRI, and other imaging examinations. The patient inclusion criteria were as follows: (1) breast cancer diagnosed on the basis of preoperative biopsy or intraoperative frozen section pathology; (2) TNM stage of cT1–2N0M0, determined according to physical and imaging examination results. The exclusion criteria were as follows: (1) a history of radiation or surgery in the affected armpit or chest wall; and (2) allergic to tracer. In total, 123 patients who met the aforementioned criteria were included in this study. Their age range was 32–81 years, with a median age of 55 years. All patients were women, 121 of them were treated for the first time and 2 were treated after NAC. The NAC protocol was “doxorubicin d1 + cyclophosphamide d1 + albumin paclitaxel d2/Q21d, a total of four cycles”. All patients signed the relevant informed consent form, which was reviewed by the Ethics Committee of Chengde Central Hospital.

### Tracer application method

**Figure 1:**
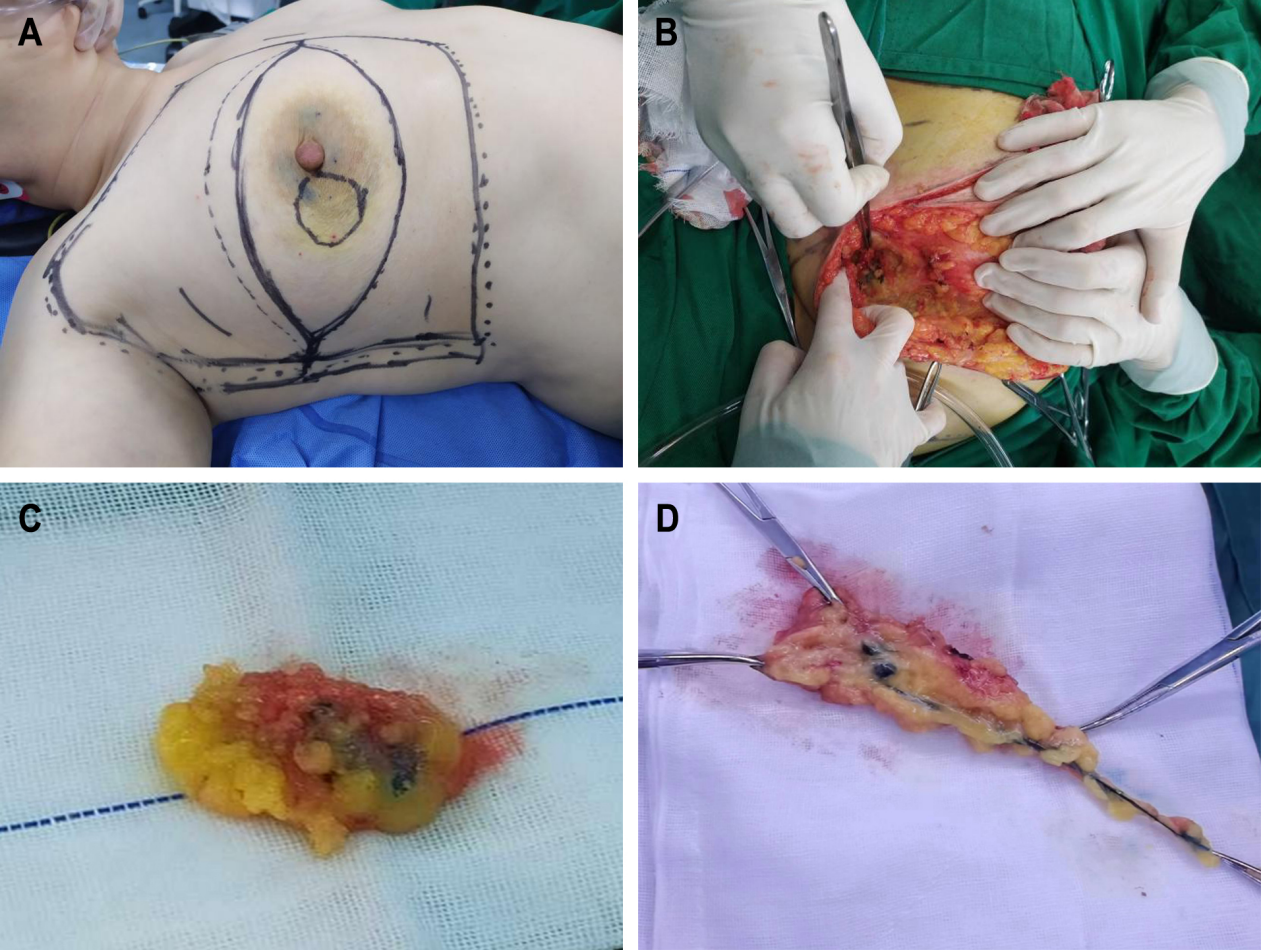
Surgical images. (**A**) Tracers were injected around the edge of areola before operation. (**B**) Color rendering of CNP in SLN can be seen during operation. (**C**) Resected CNP stained SLN and surrounding tissues. (**D**) Resected CNP stained lymph vessels, SLN and surrounding tissues.

**MB:** 1% (20 mg: 2 ml) MB injection was produced by Jichuan Pharmaceutical Group Co., Ltd. One milliliter of the original solution was taken and 0.2 ml was injected at the edge of the affected breast areola at an average interval at four places 15 min before the operation, in the same area as the ICG or CNP injected before the operation (Figure 1A).

**ICG:** 25 mg/bottle of ICG was produced by Dandong Yichuang Pharmaceutical Co., Ltd. for injection. ICG was prepared into 0.5 mg/ml solution with physiological saline, and 0.2 ml was injected at the edge of the affected breast areola at an average interval at four places 30 min before the operation. The light in the operating room was turned off, the affected breast and armpit are irradiated with a near-infrared fluorescence imaging instrument, and the lymphatic vessels and lymph nodes were marked with a marker pen.

**CNP:** 5% (50 mg: 1 ml) CNP suspension injection was produced by Chongqing Laimei Pharmaceutical Co., Ltd. One milliliter of the original solution was prepared and 0.2 ml was injected at the edge of the affected breast areola at an average interval at four places at 4–8 h before the operation.

### Operation process

Before the operation, the tracer was injected according to the aforementioned method. The patient slept in a supine position; the affected limb was abducted; and routine anesthesia, disinfection, and sheet lying were performed. The patients diagnosed as having breast cancer underwent SLNB. Those diagnosed with no breast cancer first underwent tumor resection, and the resected tumor was then sent for frozen section pathology. SLNB was performed after the pathological report of breast cancer was obtained. Following SLNB, the removed lymph nodes and surrounding tissues were sent for pathological examination, and the operation type was determined according to the pathological results. After surgery, tumor and lymph node samples were sent for pathological examination.

The SLNB operation process was as follows:

(1) Non-tracking group: The soft tissue was cut 1 cm above the anterior axillary fold. Then, the skin flap to the anterior axillary fold and the soft tissue were cut. Sharp separation was continued, and the lateral edge of the pectoralis major muscle was directly reached. The soft tissue along the lateral edge to the opposite direction of the axilla was cut, and the lymph nodes within this range were carefully searched. The lymph nodes stained by the tracer were searched, and the lymph nodes and surrounding tissues were removed (Figures 1B,C).
(2) Tracing group: The soft tissue was cut 1 cm above the anterior axillary fold. The flap toward the nipple was made free. The stained lymphatic vessels in the soft tissue were searched. The stained lymph nodes along the route of the stained lymphatic vessels were separated and searched, and the lymphatic vessels, lymph nodes, and surrounding tissues were removed (Figure 1D).

### Observation indicators

Clinical data evaluated were age, menopause status (yes or no), tumor location, maximum diameter of tumor, tumor-related immunohistochemistry, etc.

Based on different tracers, patients were divided into three groups: CNP group (38 cases), CNP + MB group (41 cases), and ICG + MB group (44 cases). The SLN identification rate, the number of SLNs detected, the false-negative number, the staining of lymphatic vessels during operation, and the tracer-related side effects or adverse reactions after operation were observed in each group. SLN identification rate = number of SLN cases detected per total number of cases * 100%. The ideal display rates of lymphatic vessels = case number of ideal lymphatic vessel display/total number of cases The false-negative number is the number of SLN-negative cases as indicated by intraoperative frozen section pathology, but the false-positive number is the number of ALN-positive cases as indicated by postoperative paraffin pathology. Staining of lymphatic vessels visible during operation or in the cut tissue is considered lymphatic vessel coloration. The tracer-related side effects or adverse reactions that were focused on include headache, dizziness, vasculitis, skin necrosis, dermatitis, skin staining, urine staining, laser irradiation injury caused by ICG identification instrument, etc.

Grouped according to the SLNB operation mode: the number of SLNs detected, the number of SLN-positive cases, the number of false-negative cases, the operation time of SLNB, and the number of adverse reactions after operation in each group. From the beginning of SLNB surgery to the removal of all SLNs and surrounding tissues, the SLNB surgery duration was recorded. The adverse reactions related to surgery included limb pain, limited activity, lymphedema, etc.

### Statistical methods

SPSS 25.0 statistical software was used. The number of SLN detected and the operation duration are expressed by mean ± standard deviation (x ± s). Multi-group mean comparison was used for the analysis of variance. The mean value of the two groups was compared using t-test. Count data, such as SLN positive number, SLN false-negative number, staining tube color number, and complications, do X^2^ Inspection. *P* < 0.05 was considered statistically significant.

## Results

### Baseline characteristics of each group applying different tracers are consistent

Multiple clinical factors, such as age, menopause status (yes or no), and tumor location, are closely related to breast cancer prognosis. To illustrate the comparability between the groups, this study first conducted statistics on the age, menopause status (yes or no), tumor location, tumor location quadrant, and other general data of the three observation groups, and the results exhibited no statistical difference (*P* > 0.05), (Supplementary Table S1).

**Figure 2:**
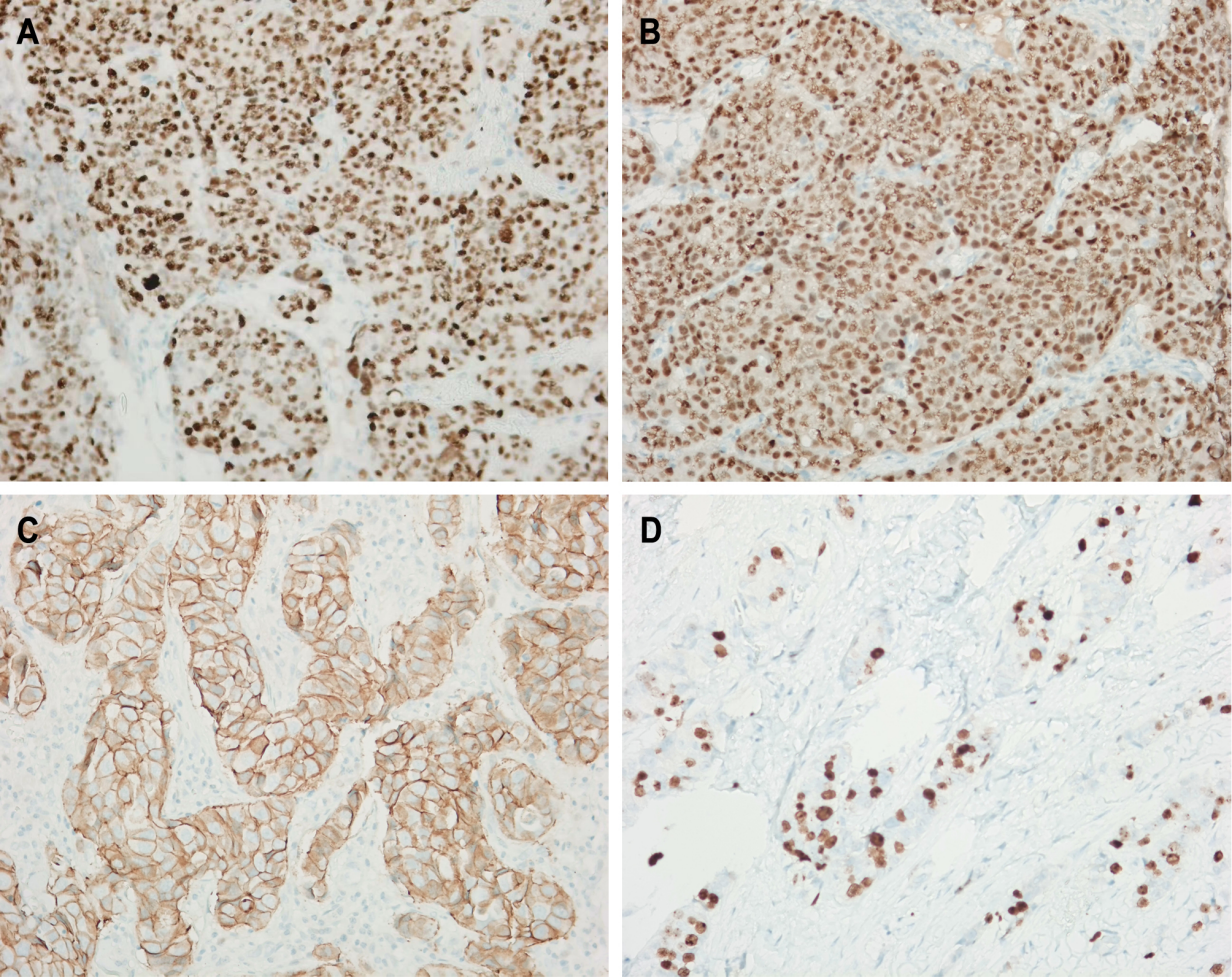
Typical immunohistochemical maps. (**A**) ER; (**B**) PR; (**C**) HER2; (**D**) Ki67.

Tumor size and immunohistochemistry are also related to tumor prognosis. The pathological data of tumor size, ER (estrogen receptor), PR (progesterone receptor), HER2 (human epidermal growth factor receptor 2), Ki67, and molecular typing results of the three observation groups were statistically analyzed. The results demonstrated no statistical difference (*P* > 0.05), (Supplementary Table S2), which indicated that each group was comparable in pathology. Typical immunohistochemical maps of ER, PR, HER2 and Ki67 are shown (Figure 2).

### Identification of SLN in each group using different tracers

The SLN identification rates in the CNP, CNP + MB, and ICG + MB groups were 37/38 (97.4%), 40/41 (97.6%), and 42/44 (95.5%), respectively (*P* > 0.05). The average number of SLNs in the three groups detected was 4.92 ± 2.06, 5.12 ± 2.18, and 4.57 ± 1.90, respectively (*P* > 0.05). One case was proved to be false negative through paraffin pathology after operation in each group (*P* > 0.05) (Table 1).

**Table 1:** Comparison of the results of SLN identification in the three groups of breast cancer patients.

Groups	*N*	SLN identification rate	Average number of SLN identification	False negative number
CNP	38	37/38 (97.4%)	4.92 ± 2.06	1
CNP + MB	41	40/41 (97.6%)	5.12 ± 2.18	1
ICG + MB	44	42/44 (95.5%)	4.57 ± 1.90	1

CNP, carbon nanoparticles; MB, methylene blue; ICG, indocyanine green; SLN, sentinel lymph node.

### Color rendering of lymphatic vessels in each group using different tracers

The lymphatic display facilitates surgeons to find SLN along the lymphatic vessels. When lymphatic vessels need not be removed, unnecessary lymphatic injury can also be avoided. The ideal color rendering rates of lymphatic vessels in the CNP, CNP + MB, and ICG + MB groups were 86.8%, 87.8%, and 93.2%, respectively, with no significant difference (*P* > 0.05), (Table 2).

**Table 2:** The ideal display of lymphatic vessels in the three groups of study patients.

Groups	*N*	Ideal lymphatic vessel chromogenesis
CNP	38	33/38 (86.8%)
CNP + MB	41	36/41 (87.8%)
ICG + MB	44	41/44 (93.2%)

CNP, carbon nanoparticles; MB, methylene blue; ICG, indocyanine green.

### Related adverse events and safety evaluation of each group using different tracers

In all 123 patients, urine blue staining occurred in the CNP + MB and ICG + MB groups, which is a side effect of MB application. The urine color in all patients became normal color within 24 h. One case and 3 cases of skin inflammation were reported in the CNP + MB and ICG + MB groups, respectively, and skin inflammation recovered after several days. In addition, no skin or tissue necrosis, nausea, vomiting, headache, urticaria, and other adverse reactions related to the tracer were observed.

### Baseline characteristics of the two groups using different SLNB surgical methods were consistent

All 123 patients were divided into two groups according to different SLNB operation methods they underwent: 53 patients in the non-tracking group and 70 patients in the tracking group. General data of the two groups including age, menopause status (yes or no), tumor location, tumor location quadrant, and axillary dissection were statistically analyzed. The results exhibited no statistical significance (*P* > 0.05), (Supplementary Table S3). The pathological data including tumor size, ER, PR, HER2, Ki67, and molecular typing results of the two groups were statistically analyzed. The results exhibited no statistically significant difference (*P* > 0.05), (Supplementary Table S4).

### No difference was observed in the application of tracer between the two groups with different SLNB operation methods

CNP was selected in 17 and 21 cases in the non-tracking and tracking groups. CNP + MB was selected in 13 and 28 cases and ICG + MB was selected in 23 and 21 cases, respectively (*P* > 0.05), (Table 3).

**Table 3:** Comparison of tracers between the non-tracking and tracking groups.

Groups	*N*	CNP	CNP + MB	ICG + MB
Non-tracking	53	17	13	23
Tracking	70	21	28	21

CNP, carbon nanoparticles; MB, methylene blue; ICG, indocyanine green.

### SLN identification and lymph node metastasis in two groups with different SLNB operation methods

The SLN identification rates in the non-tracking and tracking groups were 51/53 (96.2%) and 68/70 (97.1%), respectively (*P* > 0.05). The average number of SLNs detected in the two groups was 4.81 ± 2.12 and 4.90 ± 1.99, respectively (*P* > 0.05). The lymphatic metastasis rates in the two groups were 24.5% and 18.6%, respectively (*P* > 0.05). The number of false negatives in the two groups was 2 and 1, respectively (Table 4).

**Table 4:** Identification and metastasis of SLN.

Groups	SLN identification rate	Number of SLN	SLN metastasis		False negative
			+	−	
Non-tracking	51/53 (96.2%)	4.81 ± 2.12	13	40	2
Tracking	68/70 (97.1%)	5.70 ± 1.93	13	57	1

SLN, sentinel lymph node.

### Operation duration of two groups of SLNB with different operation methods

**Figure 3:**
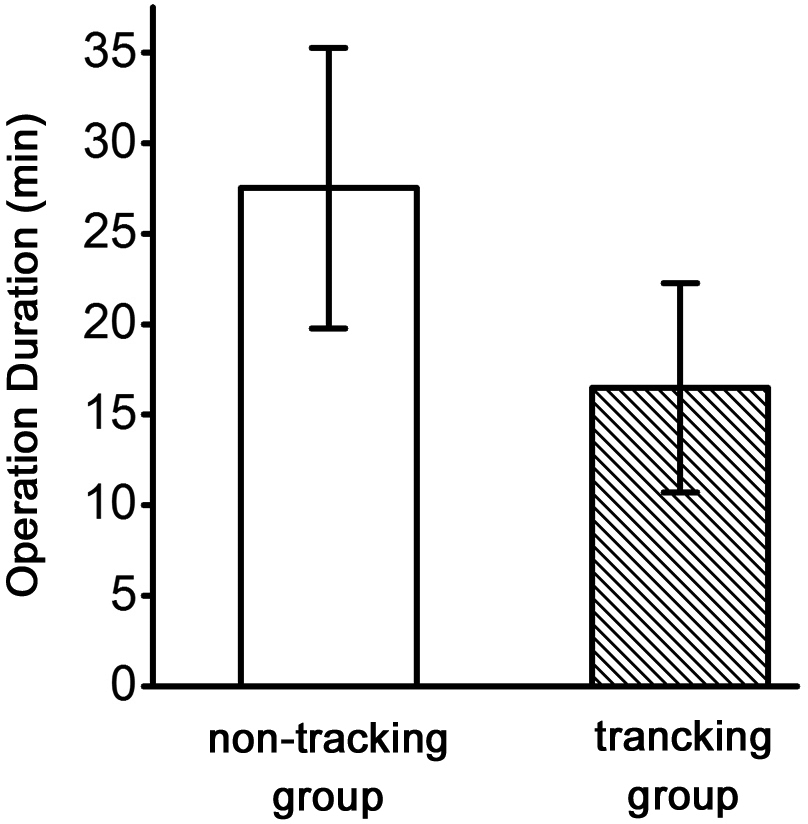
Comparison of SLNB operation duration between the non-tracking and tracking groups.

The average operation duration for the non-tracking and tracking groups was 16.47 ± 5.78 min and 27.53 ± 7.75 min, respectively. The average operation duration of the non-tracking group was 11.06 min shorter than that of the tracking group (*P* < 0.01), (Figure 3).

### Postoperative complications related to two groups of operation with different SLNB operation methods

The follow-up period was 1 month following operation. Of the 123 patients in the two groups, 118 patients were followed up, including 51 patients in the non-tracking group and 67 patients in the tracking group. Only one patient developed upper limb edema in the non-tracking group and 2 patients developed upper limb edema in the tracking group. Both groups had no sensory abnormalities and dyskinesia on the affected side (*P* > 0.05), (Table 5).

**Table 5:** Comparison of the upper limb pain after SLNB.

	*N*	Upper limb edema	
		+	−
Non-tracking	51	1	50
Tracking	67	2	65

## Discussions

This study retrospectively analyzed the clinical data of 123 primary breast cancer patients who received SLNB in our hospital from November 2017 to December 2022. Three types of tracers were used in the SLNB process, namely CNP, ICG, and MB. Among them, MB was combined with the former two and three groups of patients were formed: CNP, CNP combined with MB, and ICG combined with MB groups. No difference was observed in the SLN number and identification rates in each group, and the ideal display rate of lymphatic vessels in each group was 86.8%–93.2%. After the SLNB operation process was simplified, no effect was observed on the SLN identification number and identification rates, but the operation duration was shortened from 27.53 ± 7.75 min to 16.47 ± 5.78 min, reducing 11.06 min.

### Clinically, a considerable part of ALND is gradually replaced by SLNB

In recent years, because of the improvement in women's awareness about breast cancer, the gradual improvement in relevant screening projects, and the development of imaging technology, breast cancer is often observed and diagnosed at an early stage. The number of cases of early breast cancer in all breast cancer cases is increasing. In all cN0 patients, only approximately 25%–30% of SLN are positive (26, 27).

SLN is the first lymph node to receive lymphatic drainage from the breast, and so, SLN has the highest probability of being affected. Upper limb edema occurs after ALND operation mainly because of the obstruction of upper limb lymphatic drainage caused by the destruction of upper limb lymphatic vessels (28). If SLN transfer is absent, the possibility of other ALN transfer is very low. In this case, ALND can be avoided. Presence or absence of lymph node metastasis is the most important indicator of early breast cancer treatment (29). SLNB involves fewer lymph nodes and fewer complications than standard ALND (30, 31). SLNB is considered a reference for lymph node staging of early breast cancer (32, 33) and has been extensively known in ALN staging of early breast cancer.

The current SLN identification methods are based on staining, radionuclide method, etc. MB, ICG, CNP, radionuclide, or some combination of tracers can be used to identify SLN (21, 34, 35). When a single dye is used for SLNB, the identification rate is only 70%–86% (36, 37). Finding unstained lymphatic vessels is very time-consuming and laborious. This study presented the situation of SLNB for breast cancer patients in our hospital, which is consistent with the current development trend and reported our innovative improvement in SLNB.

### SLN identification is related to many factors

**Age:** Some studies have found that (38) the SLNB success rate of patients aged ≥60 years was significantly lower than that of patients aged <60 years. The reasons possibly were the atrophy of tissue cells in the elderly people, reduction in the degree of relaxation and tension, low hydrostatic pressure of lymphatic vessels, and slow speed of the tracer passing through lymphatic vessels.

**Menopause status:** Although no evidence is available to exhibit the impact of menopause on SLNB, menopause has an impact on the incidence of breast cancer is certain. Before and after menopause, endocrine dysfunction and hormone level disorder increase the incidence of breast cancer.

**Quadrant of tumor location:** Some studies have found that (39), when MB is used for SLNB of breast cancer and when the tumor is located in the outer upper and outer lower quadrants, the identification rate is higher than that in other quadrants, and the false-negative rate of SLN in the inner upper quadrant is higher than that in the outer upper and outer lower quadrants.

**Tumor size:** When the tumor is large, it compresses the surrounding lymphatic vessels, thereby blocking the lymphatic return. Tumor thrombi may be formed, which may block lymph vessels and lead to SLNB failure.

**Immunohistochemical indices (ER, PR, HER2, and Ki67):** Clinically, patients with high PR- and ER-positive rates exhibit good effects of endocrine therapy. HER2 is a risk factor for ALN metastasis in breast cancer. The higher the Ki67-positive rate, the higher the proliferative activity. When ER, PR, and HER2 are positive at the same time, the lymph node metastasis rate is igh, and disease-free survival and total survival periods are short (40).

To prove the comparability among the groups, the conclusions drawn are not affected by the aforementioned factors. The age, menopause status, left and right positions of the tumor, tumor location quadrant, tumor size, and immunohistochemistry (ER, PR, HER2, Ki67, and luminal typing) were compared in each group, and no significant differences were observed. This indicates that although this is not a randomized controlled prospective study, the results were comparable among the three groups.

### MB, CNP, and ICG are common clinical tracers in China

MB exists in a dissolved state and its molecular weight is extremely small. After injection, it can enter both the lymphatic capillaries and capillaries. Although the specificity of the lymphatic system is poor, it can widely stain the lymph vessels, lymph nodes, tissue spaces and blood vessels, but it demonstrated a strong binding ability with the tissue proteins, spreads slowly in the lymphatic vessels, and displays good lymphatic blue staining effect. Chen et al. (41). reported that the incidence of unsatisfactory lymphatic vessels show was significantly higher in the CNP group than in the MB or combination group.

CNP is nanotechnology-treated activated carbon, with a particle diameter of approximately 21 nm. After suspension aids are added, CNP forms a granular suspension having a diameter of 150 nm. When the capillary endothelial space is approximately 20–50 nm, CNP cannot enter. However, when the capillary lymphatic endothelial space is approximately 500 nm, CNP can easily enter. Another factor influencing the entry into lymphatic vessels is that the pressure between tissues is greater than that in lymphatic vessels, and so, there is lymphatic tendency. After CNP is injected into the local tissue, CNP quickly enters lymphatic vessels and SLN, making them appear black and exhibits good clinical safety. As a tracer for SLNB during breast cancer surgery, the accuracy rate of CNP was 96.4%, and the false-negative rate was 11.1% (42).

ICG is a green powder, whose solution is excited by infrared light (760 nm) and produces near-infrared fluorescence. Using imaging equipment, SLN can be observed to emit near-infrared light in the 750–950-nm wavelength range. The lymphatic drainage pathway and SLN position can also be observed, which can be used as an SLN tracer (43, 44). Due to the high transparency of ICG fluorescence in living tissues, it can achieve percutaneous visualization and real-time intraoperative imaging, making it easier to recognize SLN. However, the fluorescence penetration is not ideal (<1 cm). Moreover imaging deep lymph nodes is difficult, and expensive imaging equipment is required with ICG. ICG is forbidden for patients with liver insufficiency and iodine allergy.

These three commonly used tracers were selected for breast cancer SLNB in the present study.

### Combined application of the two tracers has advantages

**MB combined with CNP:** Compared with CNP, MB has strong affinity with tissues, moves slowly in lymphatic vessels, and displays lymphatic vessels better. However, it displays relatively poor color of lymph nodes. CNP swims faster in the lymphatic vessels. When SLN is colored, the color of the lymphatic vessels fades. The interval between injection and surgery is crucial. During the SNLB operation, SLN was found to be black, but lymphatic vessels were not stained. The CNP + MB combination can stain the lymphatic vessels and lymph nodes at the same time, thereby conferring complementary effects and thus reducing the difficulty associated with surgery and shortening the operation time.

**MB combined with ICG:** The disadvantage of ICG is that ICG-stained material needs to be observed under a near-infrared imager and cannot be observed with naked eyes under common light, which is inconvenient for surgery. MB's visibility characteristics can compensate for this disadvantage of ICG. The ICG + MB combination can realize the complementarity of advantages and disadvantages.

This study adopted two combinations, namely CNP combined with MB and ICG combined with MB, which achieved satisfactory SLN identification. CNP is widely employed in China, and a considerable number of medical institutions combined it with MB, although both are visible dyes. Chen et al. (41). reported that the combination of CNP and MB had a higher ideal lymphatic vessel display ratio relative to that of CNP alone. However, our results did not suggest that the addition of MB increased display rate of the lymphatic vessels. There may have been a slight difference in the degree of color, but the difference was not significant. Our study findings involved injecting CNP 4–8 h before surgery, which was undertaken in the early morning. However, Chen et al.'s report did not indicate the time of CNP injection. We believe that the possible reason for this difference is the different time intervals between injection of CNP and surgery.

However, attention should also be focused on the possible MB-induced skin inflammation. This study found 1 and 3 cases of skin inflammation in the CNP + MB and ICG + MB groups, respectively. Especially in breast-conserving surgery, CNP tracing alone can reduce the risk of skin inflammation and meet the patients' requirements for appearance.

### Identification efficiency of three tracers for SLB

Many studies have reported about the use of the three tracers in SLNB of breast cancer. Many reports about the use of CNP or ICG in combination with MB, including many comparative observations, have been published. The reported identification rate of SLN were 99.59% (135/136) (45), 99.1% (329/332) (46), 100% (24/24) (42), 98.5% (64/65) (47) using CNP, and 97.6% using MB + CNP (41). A meta-analysis of 49 studies revealed that the identification rate of breast cancer SLN using MB + ICG were 97% (22). However, a report on the tracking results of CNP + MB and ICG + MB is lacking.

This study compared 123 cases, and no statistical difference was observed in the SLN number and identification rate among the three groups. One false-negative case of SLN was reported in each of the three groups. Because the number of ALN-positive cases was small, statistical calculation cannot be performed.

The author believes that under the medical model of “early identification, early diagnosis, and early treatment,” breast cancer can often be diagnosed at an early stage. The number of metastatic cases of ALN is gradually decreasing, and most SLN-negative patients no longer undergo ALND. Therefore, determining whether cancer cell metastasis has occurred in ALN is impossible. Consequently, the amount of this experimental data was insufficient to meet the sample size required for the test. The number of cases needs to be increased for further observation.

### Lymphatic vessel imaging is convenient for finding SLN along lymphatic vessels

There are only a few reports on the display of lymphatic vessels in the study of tracers. Ye et al. (25) observed the display of lymphatic vessels and lymph nodes under different concentrations of ICG and found that the ICG + MB dual-tracer method could improve the display rate of the lymphatic vessels and lymph nodes at appropriate concentrations. In fact, as long as the dosage is sufficient and the lymphatic vessels are unobstructed, any commonly used tracer in clinical practice displayed the lymphatic vessels, but the key factor was the need for an appropriate observation time. In this study, two combinations of two tracers were studied. In the CNP, CNP + MB, and ICG + MB groups, the ideal display rates of lymphatic vessels were 86.8%, 87.8%, and 93.2%, respectively.

### Simplify SLNB procedures

For cases with unstained lymphatic vessels, finding lymphatic vessels during the operation is difficult. Finding SLN along the lymphatic vessels according to the surgical method recommended in the guide is also difficult (24).

This study simplified the SLNB operation process. According to our experience, separating and finding lymph nodes along the lymphatic path are not essential. After the tracer is injected, the soft tissue must be cut 1 cm above the anterior axillary fold. Regardless of whether the stained lymph vessels are seen or not, sharp separation must be continued, and the lateral edge of the pectoralis major muscle must be reached. The soft tissue along the lateral edge to the opposite direction of the armpit must be disconnected. In this range, the lymph nodes stained by the tracer can be found. The lymph node and some surrounding tissues should be excised to achieve satisfactory results. Generally, missed identification does not occur, which has no effect on the results of intraoperative frozen section pathology and postoperative paraffin pathology. After simplification, no effect was observed on the SLN identification number, SLN identification rate, and SLN metastasis identification rate, but the operation time was significantly shortened, with an average of 27.53 ± 7.75 min in the tracking group and 16.47 ± 5.78 min in the non-tracking group; thus, a decrease of 11.06 min was observed. After simplifying the process, the time-consuming and laborious step of finding lymphatic vessels was omitted. The operation time was reduced and the difficulty of operation was reduced. In theory, the occurrence of surgical complications can be reduced.

Surgical injury can make a few patients experience upper limb pain, upper limb edema, motor dysfunction, etc. The possible postoperative complications may be further reduced by reducing the operation steps. However, owing to the low incidence, the change in the incidence of complications in the two groups was not observed in this study.

There are several factors that affect the operation duration, such as the patient's physical condition and the presence or absence of anatomical variation; the proficiency level of the doctors; unexpected circumstances occurring during surgery; proficiency of teamwork, among others. In this study, except for the patient's uncontrollable physical condition, all surgeries were performed by the same team.

## Conclusion

This study is the first to observe the application effect of CNP combined with MB and ICG combined with MB in SLNB of breast cancer. No significant difference was observed between them in SLN identification and lymphatic vessel display. In SLNB operation, ignoring lymphatic vessels and directly looking for stained lymph nodes have the same effect as standard operation in SLN identification. However, reducing operation steps can reduce operation time and theoretically reduce postoperative complications.

## Data Availability

The original contributions presented in the study are included in the article/**Supplementary Material**, further inquiries can be directed to the corresponding author/s.

## References

[1] Noguchi M Inokuchi M Yokoi-Noguchi M Morioka E Haba Y. Conservative axillary surgery is emerging in the surgical management of breast cancer. Breast Cancer. (2023) 30(1):14–22. 10.1007/s12282-022-01409-2 36342647

[2] Thompson JL Wright GP. Contemporary approaches to the axilla in breast cancer. Am J Surg. (2023) 225(3):583–7. 10.1016/j.amjsurg.2022.11.036 36522219

[3] Cabanes PA Salmon RJ Vilcoq JR Durand JC Fourquet A Gautier C Value of axillary dissection in addition to lumpectomy and radiotherapy in early breast cancer. The breast carcinoma collaborative group of the institut curie. Lancet. (1992) 339(8804):1245–8. 10.1016/0140-6736(92)91591-U 1349666

[4] Sosa JA Diener-West M Gusev Y Choti MA Lange JR Dooley WC Association between extent of axillary lymph node dissection and survival in patients with stage I breast cancer. Ann Surg Oncol. (1998) 5(2):140–9. 10.1007/BF02303847 9527267

[5] Kim T Giuliano AE Lyman GH. Lymphatic mapping and sentinel lymph node biopsy in early-stage breast carcinoma: a metaanalysis. Cancer. (2006) 106(1):4–16. 10.1002/cncr.21568 16329134

[6] Schrenk P Rieger R Shamiyeh A Wayand W. Morbidity following sentinel lymph node biopsy versus axillary lymph node dissection for patients with breast carcinoma. Cancer. (2000) 88(3):608–14. 10.1002/(SICI)1097-0142(20000201)88:3<608::AID-CNCR17>3.0.CO;2-K 10649254

[7] Ollila DW Brennan MB Giuliano AE. The role of intraoperative lymphatic mapping and sentinel lymphadenectomy in the management of patients with breast cancer. Adv Surg. (1999) 32:349–64. 9891752

[8] Blamey RW Hornmark-Stenstam B Ball G Blichert-Toft M Cataliotti L Fourquet A ONCOPOOL—a European database for 16,944 cases of breast cancer. Eur J Cancer. (2010) 46(1):56–71. 10.1016/j.ejca.2009.09.009 19811907

[9] Carter CL Allen C Henson DE. Relation of tumor size, lymph node status, and survival in 24,740 breast cancer cases. Cancer. (1989) 63(1):181–7. 10.1002/1097-0142(19890101)63:1<181::AID-CNCR2820630129>3.0.CO;2-H 2910416

[10] Hindie E Groheux D Brenot-Rossi I Rubello D Moretti JL Espie M. The sentinel node procedure in breast cancer: nuclear medicine as the starting point. Journal of nuclear medicine: official publication. Soc Nucl Med. (2011) 52(3):405–14. 10.2967/jnumed.110.081711 21321267

[11] Giuliano AE Kirgan DM Guenther JM Morton DL. Lymphatic mapping and sentinel lymphadenectomy for breast cancer. Ann Surg. (1994) 220(3):391–8; discussion 8–401. 10.1097/00000658-199409000-00015 8092905 PMC1234400

[12] Krag DN Weaver DL Alex JC Fairbank JT. Surgical resection and radiolocalization of the sentinel lymph node in breast cancer using a gamma probe. Surg Oncol. (1993) 2(6):335–9; discussion 40. 10.1016/0960-7404(93)90064-6 8130940

[13] Veronesi U Viale G Paganelli G Zurrida S Luini A Galimberti V Sentinel lymph node biopsy in breast cancer: ten-year results of a randomized controlled study. Ann Surg. (2010) 251(4):595–600. 10.1097/SLA.0b013e3181c0e92a 20195151

[14] Krag DN Anderson SJ Julian TB Brown AM Harlow SP Costantino JP Sentinel-lymph-node resection compared with conventional axillary-lymph-node dissection in clinically node-negative patients with breast cancer: overall survival findings from the NSABP B-32 randomised phase 3 trial. Lancet Oncol. (2010) 11(10):927–33. 10.1016/S1470-2045(10)70207-2 20863759 PMC3041644

[15] Morrow M. It is not always necessary to do axillary dissection for T1 and T2 breast cancer--point. Cancer Res. (2013) 73(24):7151–4. 10.1158/0008-5472.CAN-13-1888 24347230

[16] Donker M van Tienhoven G Straver ME Meijnen P van de Velde CJ Mansel RE Radiotherapy or surgery of the axilla after a positive sentinel node in breast cancer (EORTC 10981-22023 AMAROS): a randomised, multicentre, open-label, phase 3 non-inferiority trial. Lancet Oncol. (2014) 15(12):1303–10. 10.1016/S1470-2045(14)70460-7 25439688 PMC4291166

[17] Giuliano AE Hunt KK Ballman KV Beitsch PD Whitworth PW Blumencranz PW Axillary dissection vs no axillary dissection in women with invasive breast cancer and sentinel node metastasis: a randomized clinical trial. JAMA. (2011) 305(6):569–75. 10.1001/jama.2011.90 21304082 PMC5389857

[18] Mamtani A Barrio AV King TA Van Zee KJ Plitas G Pilewskie M How often does neoadjuvant chemotherapy avoid axillary dissection in patients with histologically confirmed nodal metastases? Results of a prospective study. Ann Surg Oncol. (2016) 23(11):3467–74. 10.1245/s10434-016-5246-8 27160528 PMC5070651

[19] Sanchez AM Terribile D Franco A Martullo A Orlandi A Magno S Sentinel node biopsy after neoadjuvant chemotherapy for breast cancer: preliminary experience with clinically node negative patients after systemic treatment. J Pers Med. (2021) 11(3):172. 10.3390/jpm11030172 PMC7998155 33801435

[20] Cabanas RM. An approach for the treatment of penile carcinoma. Cancer. (1977) 39(2):456–66. 10.1002/1097-0142(197702)39:2<456::AID-CNCR2820390214>3.0.CO;2-I 837331

[21] Jiang Y Li J Chen B Bao Y Luo C Luo Y Sentinel lymph node biopsy mapped with carbon nanoparticle suspensions in patients with breast cancer: a systematic review and meta-analysis. Front Oncol. (2022) 12:818812. 10.3389/fonc.2022.818812 35419285 PMC8995566

[22] Liu HJ Sun MS Liu LY Yu ZH Chen XX Liu Q The detection rate of methylene blue combined with another tracer in sentinel lymph node biopsy of early-stage breast cancer: a systematic review and network meta-analysis. Transl Cancer Res. (2021) 10(12):5222–37. 10.21037/tcr-21-1239 35116372 PMC8798807

[23] Niebling MG Pleijhuis RG Bastiaannet E Brouwers AH van Dam GM Hoekstra HJ. A systematic review and meta-analyses of sentinel lymph node identification in breast cancer and melanoma, a plea for tracer mapping. Eur J Surg Oncol. (2016) 42(4):466–73. 10.1016/j.ejso.2015.12.007 26853759

[24] Ye JM Guo BL Liu Q Ma F Liu HJ Wu Q Clinical practice guidelines for sentinel lymph node biopsy in patients with early-stage breast cancer: Chinese Society of Breast Surgery (CSBrS) practice guidelines 2021. Chin Med J (Engl). (2021) 134(8):886–94. 10.1097/CM9.0000000000001410 33813512 PMC8078330

[25] Ye X. Application of indocyanine green in sentinel lymph node biopsy of breast cancer and analysis of dose-response. J Shanghai Jiaotong Univ Med Sci. (2017) 37(12):1634–9. 10.3969/j.issn.1674-8115.2017.12.009?

[26] Krag DN Anderson SJ Julian TB Brown AM Harlow SP Ashikaga T Technical outcomes of sentinel-lymph-node resection and conventional axillary-lymph-node dissection in patients with clinically node-negative breast cancer: results from the NSABP B-32 randomised phase III trial. Lancet Oncol. (2007) 8(10):881–8. 10.1016/S1470-2045(07)70278-4 17851130

[27] Mansel RE Fallowfield L Kissin M Goyal A Newcombe RG Dixon JM Randomized multicenter trial of sentinel node biopsy versus standard axillary treatment in operable breast cancer: the ALMANAC trial. J Natl Cancer Inst. (2006) 98(9):599–609. 10.1093/jnci/djj158 16670385

[28] Boneti C Korourian S Diaz Z Santiago C Mumford S Adkins L Scientific impact award: axillary reverse mapping (ARM) to identify and protect lymphatics draining the arm during axillary lymphadenectomy. Am J Surg. (2009) 198(4):482–7. 10.1016/j.amjsurg.2009.06.008 19800452

[29] Heerdt AS. Lymphatic mapping and sentinel lymph node biopsy for breast cancer. JAMA Oncol. (2018) 4(3):431. 10.1001/jamaoncol.2017.4000 29167885

[30] Ashiba H Nakayama R. Computerized evaluation scheme to detect metastasis in sentinel lymph nodes using contrast-enhanced computed tomography before breast cancer surgery. Radiol Phys Technol. (2019) 12(1):55–60. 10.1007/s12194-018-00491-6 30499048

[31] Nielsen MA Bull J Culpan AM Munyombwe T Sharma N Whitaker M Preoperative sentinel lymph node identification, biopsy and localisation using contrast enhanced ultrasound (CEUS) in patients with breast cancer: a systematic review and meta-analysis. Clin Radiol. (2017) 72(11):959–71. 10.1016/j.crad.2017.06.121 28774472

[32] Esposito E Di Micco R Gentilini OD. Sentinel node biopsy in early breast cancer. A review on recent and ongoing randomized trials. Breast. (2017) 36:14–9. 10.1016/j.breast.2017.08.006 28854395

[33] Manca G Rubello D Tardelli E Giammarile F Mazzarri S Boni G Sentinel lymph node biopsy in breast cancer: indications, contraindications, and controversies. Clin Nucl Med. (2016) 41(2):126–33. 10.1097/RLU.0000000000000985 26447368

[34] Okoye CL Ezeome ER. Use of methylene blue dye for lymphatic basin mapping and sentinel lymph node biopsy in breast cancer patients in Enugu, Nigeria. Niger J Clin Pract. (2022) 25(11):1805–11. 10.4103/njcp.njcp_154_22 36412286

[35] Galimberti V Cole BF Viale G Veronesi P Vicini E Intra M Axillary dissection versus no axillary dissection in patients with breast cancer and sentinel-node micrometastases (IBCSG 23-01): 10-year follow-up of a randomised, controlled phase 3 trial. Lancet Oncol. (2018) 19(10):1385–93. 10.1016/S1470-2045(18)30380-2 30196031

[36] Hung WK Chan CM Ying M Chong SF Mak KL Yip AW. Randomized clinical trial comparing blue dye with combined dye and isotope for sentinel lymph node biopsy in breast cancer. Br J Surg. (2005) 92(12):1494–7. 10.1002/bjs.5211 16308853

[37] Sugie T Sawada T Tagaya N Kinoshita T Yamagami K Suwa H Comparison of the indocyanine green fluorescence and blue dye methods in detection of sentinel lymph nodes in early-stage breast cancer. Ann Surg Oncol. (2013) 20(7):2213–8. 10.1245/s10434-013-2890-0 23429938

[38] Gong Y Sun Q Shao J Cheng H Xia H Xiong B. Multivariate analysis of sentinel lymph node biopsy in breast cancer using blue dye methods. Tumor. (2009) 29(7):680–3. 10.3781/j.issn.1000-7431.2009.07.017

[39] Liu W Yu W Xu Q Chen J He Z Jiang Y. Methylene blue guided sentinel lymph node biopsy in 276 breast cancer patients. Int J Surg. (2009) 36(7):460–3. 10.3760/cma.j.issn.1673-4203.2009.07.01119616652

[40] Arpino G Wiechmann L Osborne CK Schiff R. Crosstalk between the estrogen receptor and the HER tyrosine kinase receptor family: molecular mechanism and clinical implications for endocrine therapy resistance. Endocr Rev. (2008) 29(2):217–33. 10.1210/er.2006-0045 18216219 PMC2528847

[41] Chen Y Lyu P He J Yang X Qiu X Gu Y. Application of carbon nanoparticles followed by microscale methylthioninum chloride in sentinel lymph node biopsy for breast cancer. Int J Surg. (2016) 43(6):386–90. 10.3760/cma.j.issn.1673-4203.2016.06.008

[42] Wu X Lin Q Chen G Lu J Zeng Y Chen X Sentinel lymph node detection using carbon nanoparticles in patients with early breast cancer. PLoS One. (2015) 10(8):e0135714. 10.1371/journal.pone.0135714 26296136 PMC4546543

[43] Pitsinis V Provenzano E Kaklamanis L Wishart GC Benson JR. Indocyanine green fluorescence mapping for sentinel lymph node biopsy in early breast cancer. Surg Oncol. (2015) 24(4):375–9. 10.1016/j.suronc.2015.10.002 26555151

[44] Shen S Xu Q Zhou Y Mao F Guan J Sun Q. Comparison of sentinel lymph node biopsy guided by blue dye with or without indocyanine green in early breast cancer. J Surg Oncol. (2018) 117(8):1841–7. 10.1002/jso.25058 29790178

[45] Yang SX Wei WS Jiang QH Zhou YF Qu W Tu JH Analysis of 246 sentinel lymph node biopsies of patients with clinical primary breast cancer by application of carbon nanoparticle suspension. J Obstet Gynaecol Res. (2018) 44(6):1150–7. 10.1111/jog.13635 29673015

[46] Zhang L Huang Y Yang C Zhu T Lin Y Gao H Application of a carbon nanoparticle suspension for sentinel lymph node mapping in patients with early breast cancer: a retrospective cohort study. World J Surg Oncol. (2018) 16(1):112. 10.1186/s12957-018-1414-6 29914538 PMC6006710

[47] Ge J Yan B Cao XC. Comparison of sentinel lymph node detection by methylene blue and carbon nanoparticle suspension injection in early breast cancer. Zhonghua Zhong Liu Za Zhi. (2011) 33(3):226–8. 10.3760/cma.j.issn.0253-3766.2011.03.016 21575525

