# Supplementary material for: Comparative observation of common tracers in sentinel lymph node biopsy of breast cancer and a study on simplifying its surgical procedure
Source: Front Surg. 2023 May 15;10:1180919. doi: 10.3389/fsurg.2023.1180919 (PMC10225584; doi:10.3389/fsurg.2023.1180919)
Supplement: Supplementary file 1 [file Datasheet1.pdf]

Supplementary materials

Table S1. Comparison of the general data of the three groups of breast cancer patients

	CNP	CNP+MB	ICG+MB	F/x^2	*P*
N	38	41	44		
Age	58.92±10.47	54.54±11.02	55.02±10.43	2.125	0.124
≤60	19	27	28	2.413	0.299
＞60	19	14	16		
Menopause				3.733	0.155
yes	30	25	27		
no	8	16	17		
Tumor location				4.870	0.088
Left	18	22	31		
Right	20	19	13		
Tumor quadrant				8.668	0.371
Outer upper	10	18	18		
outer lower	5	5	10		
inner upper	14	12	13		
inner lower	6	3	2		
Areola region	3	3	1		

CNP, carbon nanoparticles; MB, methylene blue; ICG, indocyanine green

Table S2. Comparison of the pathological data of the three groups of breast cancer patients

	CNP	CNP+MB	ICG+MB	x^2	*P*
Maximum diameter				1.432	0.489
≤2cm	22	27	31		
＞2cm	16	14	13		
ER				1.480	0.477
+	27	30	36		
-	11	11	8		
PR				0.925	0.630
+	24	27	32		
-	14	14	12		
HER2				2.557	0.279
+	8	15	15		
-	30	26	29		
Ki67*				2.225	0.329
＜30%	22	17	23		
≥30%	16	24	21		
Molecular subtype				9.195	0.165
Luminal A	19	12	19		
Luminal B	8	19	17		
HER2 overexpression	5	5	6		
Triple-negative	6	5	2		

*<30% indicate as low expression, ≥ 30% indicate as high expression. CNP, carbon nanoparticles; MB, methylene blue; ICG, indocyanine green. ER, estrogen receptor; PR, progesterone receptor; HER2, human epidermal growth factor receptor 2.

Table S3. Comparison of the baseline characteristics between the non-tracking and tracking groups

	Non-tracking group	Tracking group	t/x^2 值	*P*
Age	57.57±11.25	54.93±9.69	1.395	0.166
≤60	29	45	0.828	0.363
＞60	24	25		
Menopause			0.066	0.797
Yes	36	46		
No	17	24		
Tumor location			0.269	0.604
Left	32	39		
Right	21	31		
Tumor quadrant			3.359	0.500
Outer upper	19	27		
Outer lower	9	11		
Inner upper	14	25		
Inner lower	7	4		
Areola region	4	3		
ALND			2.915	0.088
Yes	23	20		
No	30	50		

Table S4. Comparison of the pathological characteristics between the non-tracking and tracking groups

	Non-tracking group	Tracking group	x^2	*P*
Maximum diameter			0.932	0.334
≤2cm	37	43		
＞2cm	16	27		
ER			0.207	0.649
+	39	54		
-	14	16		
PR			0.231	0.631
+	37	46		
-	16	24		
HER2			0.411	0.522
+	18	20		
-	35	50		
Ki67*			0.068	0.794
＜30%	26	36		
≥30%	27	34		
Molecular subtype			0.811	0.847
Luminal A	20	30		
Luminal B	19	25		
HER2 overexpression	7	9		
Triple-negative	7	6		

ER, estrogen receptor; PR, progesterone receptor; HER2, human epidermal growth factor receptor 2.
